# Prognostic Role of microRNA-21 in Colorectal Cancer: a Meta-Analysis

**DOI:** 10.1371/journal.pone.0080426

**Published:** 2013-11-12

**Authors:** Xiaochun Xia, Baixia Yang, Xiaogang Zhai, Xiangyang Liu, Kang Shen, Zhijun Wu, Jing Cai

**Affiliations:** 1 Department of Radiation Oncology, Nantong Tumor Hospital, Nantong, Jiangsu, China; 2 Department of Nuclear Medicine, Nantong Tumor Hospital, Nantong, Jiangsu, China; 3 Department of Science and Education, Nantong Tumor Hospital, Nantong, Jiangsu, China; Queen Elizabeth Hospital, Hong Kong

## Abstract

**Background:**

To date, many studies have shown that microRNAs (miRNA) exhibit altered expression in various cancers and may play an important role as prognostic biomarker of cancers. The present meta-analysis summarizes the recent advances in the use of microRNA-21 (miR-21) in the assessment of colorectal cancer and analyzes the prognostic role of miR-21 for survival outcome.

**Methodology/Principal Findings:**

The present meta-analysis was performed by searching PubMed through multiple search strategies. Data were extracted from studies comparing overall survival (OS) in patients with colorectal cancer who showed higher expression of miR-21 than similar patients. Pooled hazard ratios (HRs) of miR-21 for survival and 95% confidence intervals (CI) were calculated. Seven studies with a total of 1174 patients were included this meta-analysis. For overall survival (OS), the pooled hazard ratio (HR) of higher miR-21 expression in colorectal cancer was 1.76 (95% CI: 1.34–2.32, P=0.000). After elimination of heterogeneity, the pooled HR was 2.32 (95% CI: 1.82–2.97, P=0.000), which was found to significantly predict poorer survival. The subgroup analysis suggested that elevated miR-21 level and patients’ survival correlated with III/IV stage (HR=5.35, 95% CI: 3.73–7.66).

**Conclusions/Significance:**

The present findings suggest that high expression of miR-21 might predict poor prognosis in patients with colorectal cancer.

## Introduction

Colorectal cancer (CRC) is the third most common cancer in humans, its incidence lagging behind only prostate cancer in men, breast cancer in women, and lung and bronchus cancer. There are 1.2 million annual new cases worldwide. The incidence and mortality of CRC have tended to decrease in the United States, but new cases and deaths in developed countries are still much higher than developing countries. A projected 142,000 new cases of CRC will be diagnosed and approximately 51,000 will die of the disease in the United States alone in 2013. The mortality of CRC accounts for approximately 9% of all cancer deaths [1]. Although the 5-year survival rate of CRC is higher in early stage by surgical resection, the long-term survival rate and prognosis of the advanced stage patients remain poor. Although genes associated with TP53 mutations [2], KRAS mutations [3,4], BRAF mutations [4,5], and defective DNA mismatch repair (dMMR) [6] have been investigated to confirm the prognostic and survival-predictive of CRC, the clinical use of these markers requires further research.

MicroRNAs (miRNAs) are endogenous, small non-coding RNAs with a length of 18–25 nucleotides. They were and first reported in 1993 [7]. These miRNAs may regulate the translation of specific protein-coding genes [8]. Recent studies have shown revealed that overexpression of microRNA-21 (miR-21) could increase cell proliferation, migration, invasion, and survival in a variety of cancer cell lines [9,10]. miR-21 was also found to be elevated in many cancers, including breast cancer, colon cancer, lung cancer, pancreatic cancer, prostate cancer, stomach cancer, hepatocellular carcinomas, ovarian cancer, and others [11,12]. Some studies have found over-expression of miR-21 to be closely associated with poor survival outcome in various cancers [13–16]. Higher levels of miR-21 expression have been found to be predictive of cancer outcome. Some teams have carried out previous meta-analyses of the relationship between miR-21 expression and NSCLC, some solid tumors [17,18].

Although some of the studies evaluated here assessed the prognostic value of miR-21 in colorectal cancer patients, the relationship between miR-21 and colorectal cancer remains controversial because these studies involved only small study populations. This meta-analysis is the first to evaluate the relationship between miR-21 expression and survival outcome in patients with colorectal cancer.

## Materials and Methods

### Search strategy

**Figure 1:**
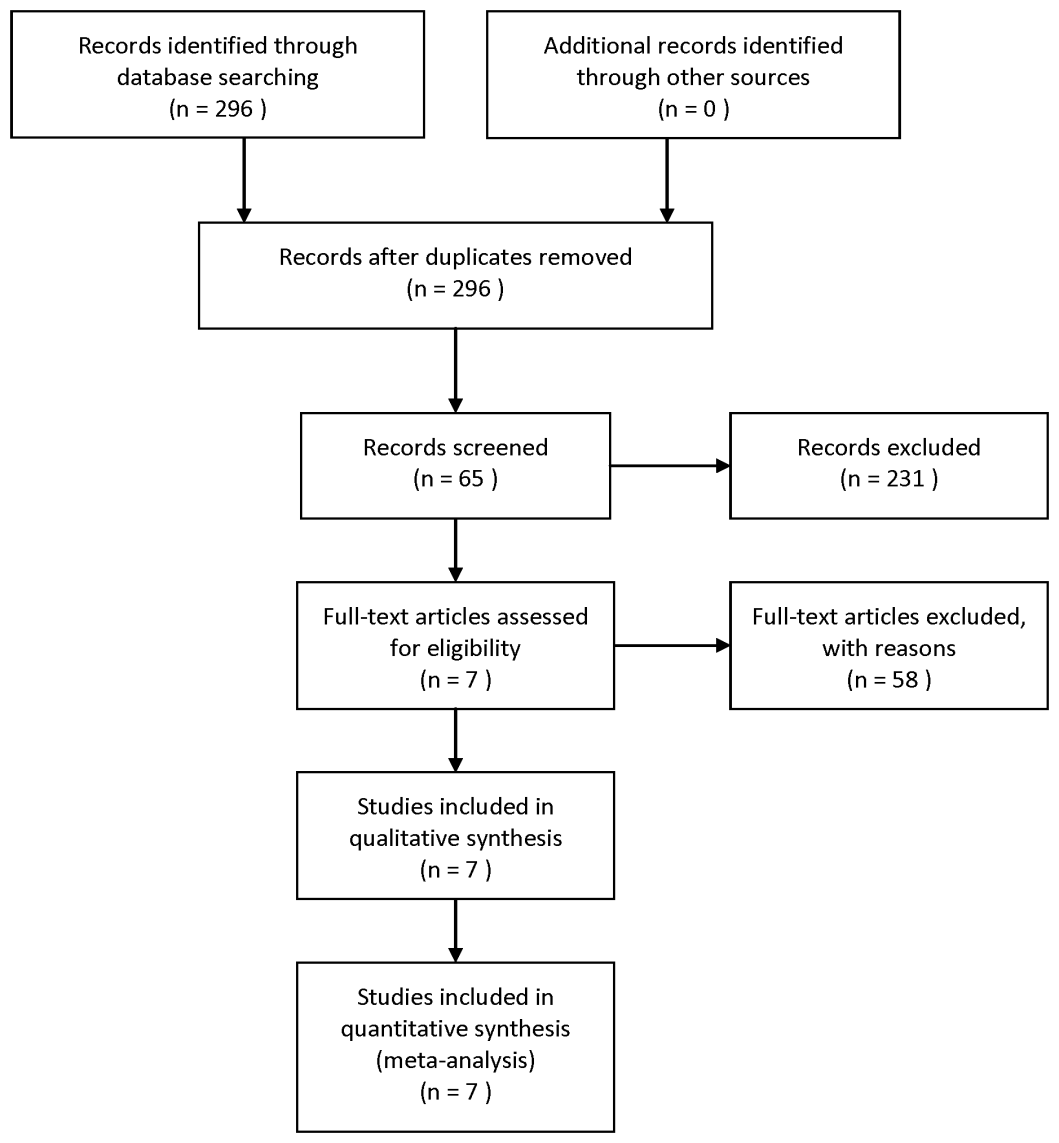
Study selection process.

This meta-analysis was carried out in accordance with the guidelines of the meta-analysis of the Observational Studies in Epidemiology group (MOOSE) [19]. The Pubmed database was searched for the last time on August, 2013, and no lower date limit was used. Only reviews published in English were evaluated. Conference abstracts were not in the scope of this analysis because of the incomplete data. The search strategy used the following terms variably combined by “microRNA-21,” ‘‘miR-21,” ‘‘colon,’’ ‘‘colorectal,” “rectum,” “cancer,” “carcinoma,” “prognosis,” and “prognostic.” Eligible studies included in this meta-analysis met the following criteria: (i) They had to discuss patients with colon cancer or rectal cancer; (ii) They had to measure the miR-21 expression in tumor tissue or serum; and (iii) They had to investigate the overall survival outcome or the correlation between miR-21 expression and the clinical variables. (iv) The method of miR-21 detection must be same. Articles were excluded based on any of the following criteria: (i) They were review articles, letters, or laboratory articles; (ii) They were not in English; (iii) They lacked key information for calculation using methods established by Parmar, Williamson, and Tierney [20–22]; (iv) They were repeated studies included the same author and the same samples from the same patients as a study already included. Two reviewers (Xiaochun Xia and Baixia Yang) independently evaluated titles and abstracts of the identified articles in duplicate. A flow diagram of the study selection process is presented in Figure 1.

### Data extraction

Eligible papers were reviewed independently by two investigators, Xiaochun Xia and Baixia Yang. Data were extracted from each study according to the before-mentioned selection criteria. Two investigators (Xiaochun Xia and Baixia Yang) extracted the primary information, including multivariate analysis, Kaplan–Meier survival analysis, *P* value, and hazard ratios independently. Further data were extracted from the studies. These included first author’s name, year of publication, origin of the study population, size of the study population, study design, type of cancer, TNM stage, sampling site, method of detecting miR-21, cutoff value, and duration of follow up. Other extracted data were reviewed including clinicopathological features (gender, age, location of the tumor, the CEA level, and other factors), HRs of miR-21 for survival, 95% confidence interval (CI) and *P* value. If only survival curves were available, data were extracted from these curves using the described method [20]. HR values >1 were considered indicative of significant associations with poor outcome. Disagreements were resolved by discussion. All the data were subject to consensus.

### Statistical methods

Heterogeneity was assessed using Q statistics (*P*<0.10 was considered heterogeneous). Any significant heterogeneity among the studies was resolved using the random-effects model. Otherwise, the fixed-effects model was used. The I^2^ statistic, which measures the percentage of the total variation across studies that is due to heterogeneity rather than to chance, was also assessed [23]. Some studies did not list the HRs or 95% CI directly, instead giving Kaplan-Meier survival curves alone. The necessary statistics were calculated using software designed by Matthew Sydes and Jayne Tierney [22]. The effect of miR-21 expression on survival outcome (OS) were estimated using forest plots. Subgroup analysis of pooled hazard ratios of colorectal cancer patients with elevated miR-21 expression were examined with respect to TNM stage (III/IV vs. I/II), gender (male vs. female), age (≥median vs. <median), tumor location (proximal vs. distal), and the CEA level (cutoff value vs. ≤cutoff value). Pooled HR was calculated using a fixed-effects model or random-effects model as appropriate. Pooled HR>1 indicated poor prognosis for the groups with elevated miR-21 expression and was considered statistically significant if the 95% CI did not overlap 1. Publication bias was evaluated using the funnel plot and Begg’s test, *P*>0.05 was considered indicative of a lack of publication bias [24]. All analyses were performed using STATA vision 10.0 (Stata Corporation, College Station, TX, U.S.). A *P* value less than 0.05 was considered to be statistically significant except where otherwise specified.

## Results

### Study characteristics

Seven studies were identified as eligible for full-text review [25–31]. These eligible studies were published between 2008 and 2013. Two studies evaluated patients from Japan, one evaluated patients from Denmark, one evaluated patients from the Czech Republic, one evaluated patients from Hong Kong, one evaluated patients from China, one evaluated patients from Taiwan, and one evaluated patients from the United States of America. These studies included a total of 1174 patients with a mean number of 167.7 patients per study. These seven eligible studies were all retrospective cohort studies. The method of miR-21 detection was all quantitative real-time polymerase chain reaction (qRT-PCR). microRNA-21 expression levels were measured in tumor tissue or serum. Six of the eligible studies carried out the univariate analysis and multivariate analysis. The mean length of follow-up ranged from 36.4 to 84.6 months. Characteristics of the eligible studies are summarized in Table 1.

**Table 1:** Characteristics of 7 retrospective cohort studies included in the present meta-analysis.

**Author**	**Origin of population**	***N***	**Disease**	**stage**	**Sampling site**	**Method**	**Cut off**	**Survival analysis**	**Hazard ratios**	**Follow-up (months)**
Schetter 2008	USA, HK	197	CC	i-iv	tumor	qRT-PCR	Highest tertile	OS	Reported	USA:68.0(26.0-141.9) HK:84.6(60.4-147.2)
Shibuya 2010	Japan	156	CRC	A-D*	tumor	qRT-PCR	Mean	OS	Reported	60
Nielsen 2011	Denmark	196	CRC	i-iii	tumor	qRT-PCR	65th percentile	OS	Reported	72
Faltejskova 2012	Czech	44	CRC	i-iv	tumor	qRT-PCR	Median	OS	SC	84
Liu 2013	China	200	CRC	i-iv	serum	qRT-PCR	--	OS	Reported	36.4(4-53)
Chen 2013	Taiwan	195	CRC	i-iv	tumor	qRT-PCR	Mean	OS	Reported	60
Toiyama 2013	Japan	186	CRC	i-iv	Serum, tumor	qRT-PCR	0.0031, 3.7	OS	Reported	60

The studies included here are all retrospective cohort studies with different groups of patients. CC, colon cancer; CRC, colorectal cancer; *, Duke’s stage; qRT-PCR, quantitative real-time PCR; -- not mentioned; OS, overall survival; SC, survival curve.

### Meta-analysis results

As shown in Table 1, six studies reported the HR and 95% CI directly, and one study did not.

**Figure 2:**
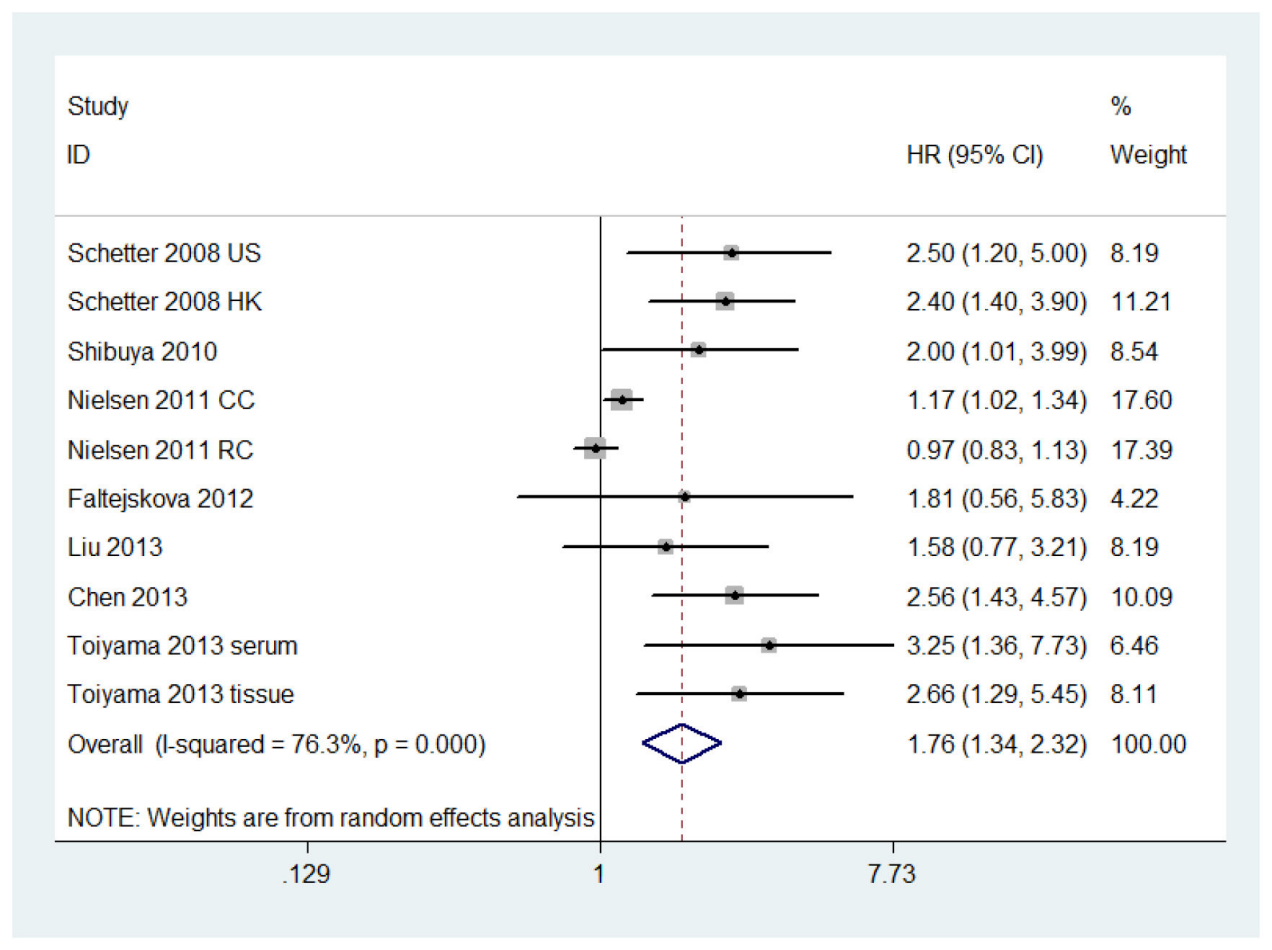
Forest plot of the relationship between elevated miR-21 level and overall survival (OS) in patients with colorectal cancer.

HR and other data were extrapolated from the Kaplan-Meier survival curves given in the eligible studies. Some heterogeneity was detected between the studies, as indicated by evaluation of the relationship between the elevated miR-21 level and OS(I^2^=76.3%, *P*=0.000). Because of this, a pooled HR and its 95% CI by random effect model were calculated. Forest plots of the individual HR estimates and the results of meta-analysis are presented in Figure 2.

**Figure 3:**
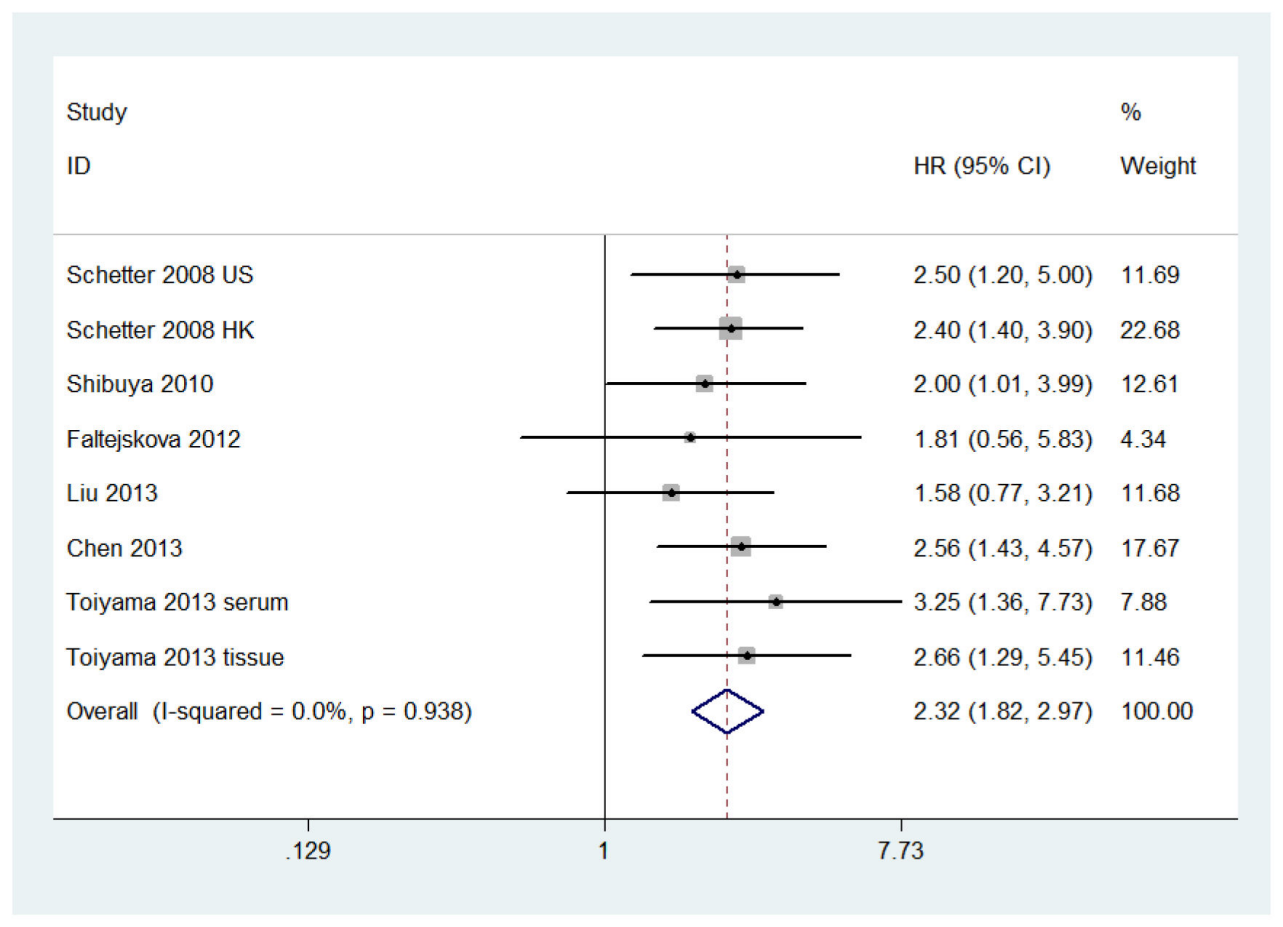
Forest plot of the relationship between elevated miR-21 level and OS among patients with colorectal cancer after elimination of heterogeneity.

According to these results, higher levels of miR-21 expression were significantly predictive of poor OS, though a significant degree of heterogeneity was observed. The pooled HR was 1.76 (95% CI: 1.34–2.32, *P*=0.000). This heterogeneity was eliminated when Nielsen’s study[27] was excluded. The pooled HR was 2.32 (95% CI: 1.82–2.97, *P*=0.000) for OS after eliminating the heterogeneity (Figure 3). We also performed subgroup analysis by TNM stage, gender, age, tumor location, and the CEA level. Heterogeneity existed in the CEA level (I^2^=84.6%, *P*=0.011) but not in other factors. The results showed that a significant relation between elevated miR-21 level and survival was also exhibited in III/IV stage (HR=5.35, 95% CI: 3.73–7.66) (Table 2).

**Table 2:** Subgroup analysis of pooled hazard ratios of colorectal cancer patients with elevated miR-21 expression.

**Subgroup analysis**	**No. of studies**	**No. of patients**	**Model**	**Pooled HR (95% CI)**	**P-value**	**Heterogeneity (I^2^, P-value)**
TNM stage (Ⅲ/Ⅳ vs. Ⅰ/Ⅱ)	4	734	fixed	5.35 [3.73–7.66]	0.000	0.0%, 0.452
Gender (male vs. female)	4	788	fixed	1.05 [0.82–1.36]	0.683	0.0%, 0.727
Age (≥median vs. <median)	4	774	fixed	1.01 [0.99–1.04]	0.370	22.0%, 0.268
Tumor location (proximal vs. distal)	2	397	fixed	0.85 [0.65–1.13]	0.263	0.0%, 0.497
CEA (cutoff value vs. ≤cutoff value)	2	386	random	2.48 [0.73–8.47]	0.146	84.6%, 0.011

**Figure 4:**
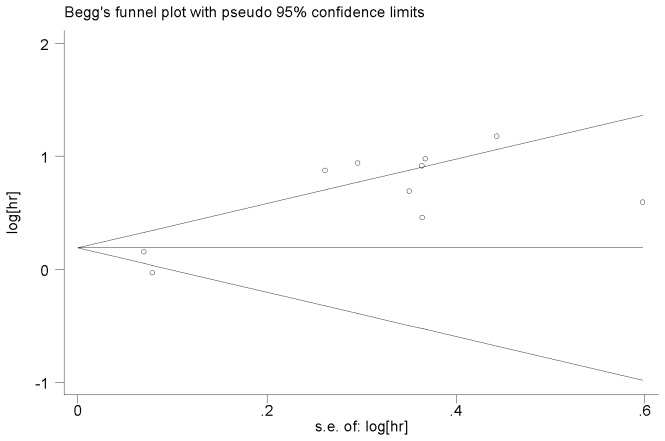
Funnel plot of high miR-21 expression and overall survival among patients with colorectal cancer.

**Figure 5:**
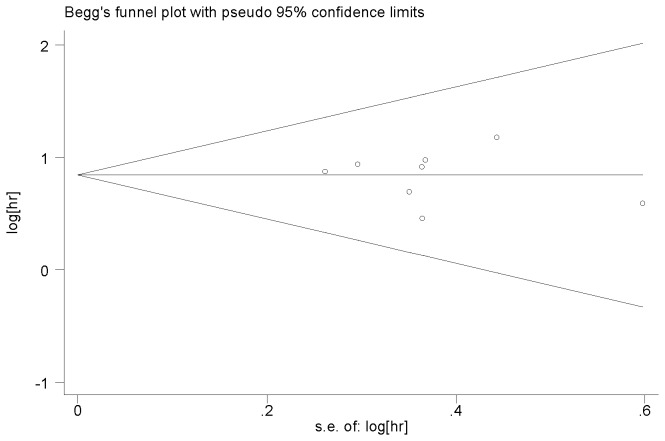
Funnel plot of high miR-21 expression and overall survival among patients with colorectal cancer after elimination of heterogeneity.

The other factors did not indicate any significant prognostic impact of higher expression miR-21. These factors included age, gender, tumor location, and the CEA level. Publication bias was evaluated using funnel plots and Begg’s tests. No significant publication biases were observed in this meta-analysis (*P*=0.788 and *P*=0.621, respectively) (Figure 4 and 5).

## Discussion

This meta-analysis is the first systematic evaluation of the relationship between miR-21 expression level and the prognosis of the colorectal cancer patients. The prognostic role of miR-21 in various cancers is still a puzzle, although some studies have discussed its role in head and neck squamous cell carcinoma (HNSCC), carcinomas of the digestion system, and non-small-cell lung cancer (NSCLC) [17,18].

Elevated miR-21 expression was found to be predictive of poor survival among colorectal cancer patients in this meta-analysis. The pooled HR of OS was 1.76 (95% CI: 1.34–2.32, *P*=0.000). The differences were found to be statistically significant, though the HRs were not strong. Hayes et al. reported that HR>2 was considered strongly predictive [32]. After elimination of heterogeneity, the pooled HR was 2.32 (95% CI: 1.82–2.97, P=0.000), which was found to significantly predict poorer survival. In the subgroup analysis, the patients’ clinical characteristics, including male gender, age, tumor location, and the CEA level showed no correlation with miR-21 expression, but III/IV stage showed significant correlations. Significant heterogeneity was observed during the meta-analysis of OS. The heterogeneity of the population may be come from differences in the clinical characteristics of patients (origin of population, tumor stage, age, etc.), the internal control, the cut-off value, the time of follow-up, or other differences. This meta-analysis was carried out using a random effect model to minimize the residual confounding effect. Heterogeneity could not be eliminated even after using random effect model. When excluding Nielsen’s study, the heterogeneity was eliminated. In Nielsen’s study, we found that the advanced patients(IV stage) were not included and this maybe lead the heterogeneity. Subgroup analysis was performed by TNM stage, gender, age, tumor location, and the CEA level to eliminate the technique bias. Begg’s test indicated no publication bias (*P*>0.05).

The biological function of miR-21 may affect the relationship between miR-21 expression and cancer outcome. miR-21 was also found to be highly expressed in many cancerous tissues including breast, colon, lung, pancreas, prostate, stomach, liver, and ovary tissues [11,12]. Recent studies have shown that miR-21 acts as an oncogene in cells and the molecular mechanism by which it regulates cellular processes has been studied [12]. Overexpression of miR-21 may increase cell proliferation, migration, invasion, and survival in a variety of cancer cell lines [9,10]. However, suppression or knock-down of miR-21 may reduce cell proliferation and invasion by inducing apoptosis [33–36]. The oncogenic role of miR-21 is showed by targeting several tumor suppressor genes including include programmed cell death 4 (PDCD4) [9,34], phosphatase and tensin homolog (PTEN) [33], cell division cycle 25 homolog A (Cdc25a) [37], reversion-inducing cysteine-rich protein with kazal motifs (RECK) [38], and tropomyosin 1 (TPM1) [39]. The relationship between PDCD4 and miR-21 is of particular interest because of the reverse association confirming in normal and cancerous tissues. Wang et al. found that miR-21 regulated G1-S transition negatively and participated in DNA damage-induced G2-M checkpoint by inhibiting Cdc25a in colon cancer cell lines [37].

The present meta-analysis had some limitations. First, there are only a few studies regarding specifically miR-21 and the prognosis of colorectal cancer. Second, TNM stage of the patients was not the same in all studies and this may have caused some of the heterogeneity observed here. Third, the cut-off values were different in various studies. Researchers defined the expression level of elevated microRNAs using cut-off values. Median and mean values were the primary cut-off values. However, there was no final conclusion that confirmed how high was considered high for these purposes. Fourth, in the present meta-analysis, elevated levels of miR-21 expression were found to have a prognostic role in colorectal cancer, but it was not possible to confirm miR-21 as independent predictive factor. Recently, researchers considered that a set of miRNAs might have a stronger predictive effect on survival than a single microRNA [40]. Fifth, the expression of miR-21 was detected in the samples of tumor tissue mostly but few in serum or plasma in the selected studies. Circulating prognostic markers were found to be more valuable than tissue throughout the lives of the cancer patients.

In summary, the present meta-analysis showed elevated miR-21 expression levels to be closely associated with poor prognosis in colorectal cancer patients. More multi-center clinical investigations with larger sample sizes should be conducted to confirm these findings.

## Supporting Information

Checklist S1 **PRISMA 2009 Checklist.**
(DOC)
